# Calcium Ions Stimulate the Hyperphosphorylation of Tau by Activating Microsomal Prostaglandin E Synthase 1

**DOI:** 10.3389/fnagi.2019.00108

**Published:** 2019-05-09

**Authors:** Long-Long Cao, Pei-Pei Guan, Yun-Yue Liang, Xue-Shi Huang, Pu Wang

**Affiliations:** College of Life and Health Sciences, Northeastern University, Shenyang, China

**Keywords:** Alzheimer’s disease, microsomal prostaglandin E synthase 1, tau, prostaglandin E_2_, EP receptors

## Abstract

Alzheimer’s disease (AD) is reportedly associated with the accumulation of calcium ions (Ca^2+^), and this accumulation is responsible for the phosphorylation of tau. Although several lines of evidence demonstrate the above phenomenon, the inherent mechanisms remain unknown. Using APP/PS1 Tg mice and neuroblastoma (N)2a cells as *in vivo* and *in vitro* experimental models, we observed that Ca^2+^ stimulated the phosphorylation of tau by activating microsomal PGE synthase 1 (mPGES1) in a prostaglandin (PG) E_2_-dependent EP receptor-activating manner. Specifically, the highly accumulated Ca^2+^ stimulated the expression of mPGES1 and the synthesis of PGE_2_. Treatment with the inhibitor of Ca^2+^ transporter, NMDAR, attenuated the expression of mPGES1 and the production of PGE_2_ were attenuated in S(+)-ketamine-treated APP/PS1 Tg mice. Elevated levels of PGE_2_ were responsible for the hyperphosphorylation of tau in an EP-1-, EP-2-, and EP-3-dependent but not EP4-dependent cyclin-dependent kinase (Cdk) 5-activating manner. Reciprocally, the knockdown of the expression of mPGES1 ameliorated the expected cognitive decline by inhibiting the phosphorylation of tau in APP/PS1 Tg mice. Moreover, CDK5 was found to be located downstream of EP1-3 to regulate the phosphorylation of tau though the cleavage of p35 to p25. Finally, the phosphorylation of tau by Ca^2+^ contributed to the cognitive decline of APP/PS1 Tg mice.

## Introduction

Tau, a family of microtubule-associated proteins, is a nervous system-specific protein that promotes microtubule assembly and stability (Cleveland et al., 1977). Structurally, tau shows little tendency for aggregation in solution as a natively unfolded or intrinsically disordered protein (Jeganathan et al., 2008). In AD, tau is reportedly hyperphosphorylated and aggregated in neurofibrillary tangles (NFTs) (Grundke-Iqbal et al., 1986). However, the mechanisms underlying tau pathology and tau-mediated neurodegeneration remain under debate. For example, hyperphosphorylated tau (p-tau) protein in cerebrospinal fluid examination (CSF) is a core biomarker candidate of AD (Buerger et al., 2006). In addition, p-tau proteins aggregate and detach from microtubules to induce the apoptosis or death of neurons (Illenberger et al., 1998; Mandelkow and Mandelkow, 1998). Moreover, p-tau proteins aggregate to form oligomer or fibrillar species, which are extremely toxic to neurons, and this aggregation leads to neuronal death (Shahani and Brandt, 2002).

Regarding the mechanism, most studies have suggested that Cdk5 is responsible for the phosphorylation of tau, and this phosphorylation destabilizes microtubules and disrupts nutrient transport in axons (Baumann et al., 1993; Reddy, 2011). Due to the critical roles of Cdk5 in tau phosphorylation, other molecules involved in regulating the activity of Cdk5, such as GSK3β, ERK1/2, c-Jun and Ca^2+^/calmodulin-dependent protein kinase II (CAMKII) (Baudier and Cole, 1988; Reynolds et al., 2000; Lucas et al., 2001; Harris et al., 2004). might also be effective in modulating the phosphorylation of tau. To address this hypothesis, previous studies have demonstrated that p25 produced from p35 via the activation of calpain is able to activate Cdk5 through a Ca^2+^-dependent mechanism (Patrick et al., 1999; Lee et al., 2000; Nath et al., 2000). In addition, p25 accumulates and activates Cdk5 in brains with AD (Tseng et al., 2002). 2-Aminothiazole, an inhibitor of Cdk5 and p25, is a potential therapeutic agent for the treatment of AD (Helal et al., 2004). Additionally, the administration of fisetin, which is a small orally active molecule that can act on the p25/Cdk5 pathway, to APP/PS1 Tg mice from 3 to 12 months of age prevents the development of learning and memory deficits (Currais et al., 2014). Therefore, p25 is potentially involved in the mechanism through which Ca^2+^ regulates the phosphorylation of tau.

Interestingly, Ca^2+^ is able to stimulate the expression of COX-2 via a calcium-sensing receptor in fibroblasts (Ogata et al., 2006). In addition, both endogenous and exogenous Ca^2+^ ions can activate COX-2 in cancer cells and osteoblasts (Wang J.Y. et al., 2012). Additionally, Ca^2+^ influx and COX-2 production have shown a similar tendency in human microglial cells (Hong et al., 2006). These findings provide evidence showing that COX-2 might be a downstream target for mediating the roles of Ca^2+^ in stimulating the phosphorylation of tau.

Consistent with the above observations, prostaglandin PGE_2_ is selectively increased in CSF at the onset of AD symptoms (Montine et al., 1999). PGE_2_ signaling through its receptors plays various roles in chronic inflammatory diseases, such as AD (Andreasson, 2010). EP1 mediates the effects of PGE_2_ on exacerbating neurotoxicity during the development and progression of AD (Zhen et al., 2012). EP2 signaling has the ability to suppress beneficial microglial functions (Johansson et al., 2015), which are responsible for inflammation in APP/PS1 Tg mice (Johansson et al., 2013). The deletion of EP2 also reduces the burden of Aβ in a model of AD (Liang et al., 2005). EP3 mediates the effects of PGE_2_ by impairing presynaptic Mf-CA3 long-term potentiation (LTP) in APP/PS1 Tg mice (Maingret et al., 2017). In addition, the deletion of EP3 attenuates the induction of proinflammatory genes, protein expression and lipid peroxidation (Shi et al., 2012). In contrast, various studies have suggested a beneficial effect of EP4 signaling on suppressing inflammation in the brain (Shi et al., 2010; Woodling et al., 2014).

Based on these findings, we showed that high concentrations of Ca^2+^ can activate inflammatory signals of COX-2 in cultured N2a cells and in an *in vivo* model. As the downstream target of COX-2, mPGES1, which is the synthase of PGE_2_, was also upregulated, and the upregulation of this protein induced the production of p25 and was thus responsible for the phosphorylation of tau. Moreover, we showed that EP1, EP2, and EP3, but not EP4, mediated the effects of PGE_2_ on the phosphorylation tau via a p25-dependent mechanism and ultimately accelerated the cognitive decline of APP/PS1 Tg mice.

## Materials and Methods

### Reagents

CaCl_2_ was purchased from Bodi Chemical Co., Ltd. (Tianjin, China). Antibodies specific against NeuN and Alexa Fluor-488, Alexa Fluor-555, and HRP-labeled secondary antibodies were purchased from Cell Signaling Technology (Danvers, MA, United States). S(+)-Ketamine (60 mg/kg, for 1 h), SC-51322 (30 nM, for 12 h), PF-04418948 (100 nM, for 12 h), and DG-041 (60 nM, for 12 h) were obtained from R&D Systems (Minneapolis, MN, United States), and CJ-42794 (40 nM, for 12 h) was obtained from MedChem Express (Monmouth Junction, NJ, United States). High-fidelity (HF) restriction enzymes for EcoRI, BamHI, XhoI, and AgeI were purchased from New England Biolabs (Beverly, MA, United States). DAPI was procured from Beyotime Institute of Biotechnology (Haimen, China). The plko.1-puro, psPAX_2_, pMD_2_.G, and plvx-IRES-zsgreen vectors were purchased from Addgene (Sidney, SD, United States). All the reagents used for the quantitative (q)RT-PCR and SDS-PAGE experiments were purchased from Bio-Rad Laboratories (Hercules, CA, United States), and all other reagents were obtained from Invitrogen (Carlsbad, CA, United States), unless otherwise specified.

### Tg Mice and Treatments

Wild-type (WT) and APP/PS1 (Stock No. 004462) mice were obtained from The Jackson Laboratory (Bar Harbor, ME, United States). In APP/PS1 Tg mice, the neurons in the brains doubly expressed a chimeric mouse/human amyloid precursor protein (Mo/HuAPP695swe) and a mutant human presenilin 1 (PS1-dE9). Both mutations are associated with early-onset AD. Tg mice showed Aβ deposition at approximately 6–7 months of age. At 9 months, APP/PS1 Tg mice exhibited obvious learning impairment compared with WT mice. COX-2 Tg mice (Stock No. 010703) were obtained from The Jackson Laboratory (Bar Harbor, ME, United States). Genotyping was performed at 3–4 weeks after birth. Five mice per cage were housed in a controlled environment with a standard room temperature, a standard relative humidity, a 12-h light/12-h dark cycle and free access to food and water. The general health and body weights of the animals were monitored daily. The brains of the mice in the different groups were collected under anesthesia and perfusion-fixed as previously described (Wang X. et al., 2010).

### Intracerebroventricular Injection (i.c.v)

CaCl_2_, lentivirus particles, or vehicles were injected (i.c.v.) into WT mice, as previously described (Yu et al., 2015; Wang et al., 2016). In select experiments, the WT mice were injected (i.c.v.) with the lentivirus particles in the absence or presence of PGE_2_. Briefly, stereotaxic injections were administered at the following coordinates relative to the bregma: mediolateral, 2.10 mm; anteroposterior, 2.00 mm; and dorsoventral, 2.28 mm. After the injection, each mouse recovered spontaneously on a heated pad. The reliability of the injection sites was validated by injecting trypan blue dye obtained from Invitrogen (Carlsbad, CA, United States) in separate cohorts of mice and observing the staining of cerebral ventricles. Twenty-four hours after injection, the mice were sacrificed under anesthesia and perfused (Yu et al., 2015; Wang et al., 2016).

### Cell Culture

Mouse neuroblastoma (N)2a cells were grown (at 37°C and 5% CO_2_) on 6-cm tissue culture dishes (1 × 10^6^ cells per dish) in appropriate medium. In a separate set of experiments, the cells were grown in serum-free medium for an additional 24 h before incubation with inhibitors in the absence or presence of CaCl_2_, as previously described (Wang P. et al., 2010, Wang P. et al., 2012; Wang et al., 2014b).

### Primary Neuron Culture

Primary cortical neurons were derived from C57BL/6 mice at embryonic day 15 and cultured for up to 21 days *in vitro*. Briefly, after the brains were dissected, the cortical hemispheres were collected, and the meninges were removed. The hippocampus was separated from the basal ganglia and cerebral cortex, minced and trypsinized (0.05% w/v) to isolate single neurons at 37°C for 20 min. The cells were then seeded in poly-L-lysine-precoated plates at a density of 1.5 × 10^5^ cells/cm^2^ and cultured with 25 μM glutamate in the medium to initiate neurite development *in vitro* for 3 days. The cells were then cultured in standard medium with 1 mL of B27/neurobasal, 0.5 mM glutamine and high concentrations of antibiotics, including penicillin, and streptomycin.

### Brain Processing

The mice were manually restrained, and deep anesthesia was induced through the intraperitoneal injection of pentobarbital sodium (100 mg/kg). At the appropriate timepoint, the level of anesthesia was checked by pinching the footpad and tail of the animals. Once the mice showed no response to footpad and tail pinching, the mice were laid with their dorsal side down, and each of the footpads were fixed to the animal operating table. The thoracic cavity was opened by cutting the ribs, and the anterior part of the rib cage was lifted to expose the heart. The animal was then perfused with oxygenated PBS(-) solution using a peristaltic pump for 2 min. The brain and the endocranium were then removed from the skull, and the brains were stored in a refrigerator at -80°C or fixed with 4% paraformaldehyde in PBS(-) before further processing.

### Quantitative Real-Time PCR

Total RNA was extracted using the TRIzol reagent (Invitrogen Carlsbad, CA, United States) and treated with DNase I (Pierce Rockford, IL, United States). The amount and purity of the RNA were determined using a NanoDrop 2000C Spectrophotometer (Thermo Fisher Scientific, Waltham, MA, United States). Quantitative real-time (qRT)-PCR assays were performed with a Mini Opticon real-time PCR detection system (Bio-Rad) using the total RNA and a GoTaq one-step real-time PCR kit with SYBR Green (Promega, Madison, WI, United States) as previously described (Yu et al., 2015). The volume and concentration of the reagents used for qPCR are presented in Table 1. The following primers were used: mPGES1 (NM_022415), F-GGATGCGCTGAAACGTGGA and R-CAGGAATGAGTACACGAAGCC; calpain (NM_001110504.1), F-GCAGGGGATGACATGGAGAT and R-CTTCCCGTTGCCATCTCGAT; EP1 (NM_013641), F-CCTCGTCTGCCTCATCCATC and R-AACACCACCAACACCAGCA; EP2 (NM_008964.4), F-GCTCCTTGCCTTTCACAATCT and R-AGGACCGGTGGCCTAAGTAT; EP3 (NM_011196.2), F-TGGTCGCCGCTATTGATAATGA and R-GCAGCAGATAAACCCAGGGA; EP4 (NM_001136079.2), F-TCATCTGCTCCATTCCGCTC and R-GGATGGGGTTCACAGAAGCA; and GAPDH (NM_001289726.1), F-AACTTTGGCATTGTGGAAGG and R-ACACATTGGGGGTAGGAACA. The gene expression levels were normalized to those of GAPDH.

**Table 1:** The volume and concentration of qPCR system.

Component	Volume	Final concentration
Go Taq qPCR Mastermix (2 × )	10 μl	1 ×
Forward primer (10 μM)	1 μl	0.5 μM
Reverse primer (10 μM)	1 μl	0.5 μM
cDNA template	0.25 μl	
Nuclease-free water	7.75 μl	


### Western Blot Analysis

Tissues were lysed in RIPA buffer (25 mM Tris–HCl (pH 7.6), 150 mM NaCl, 1% NP-40, 1% sodium deoxycholate, and 0.1% SDS) containing a protease inhibitor cocktail (Thermo Scientific-Pierce, Rockford, IL, United States) for 30 min at 4°C. The lysates were then centrifuged at 12,000 × *g* and 4°C for 20 min. The soluble protein concentrations in the lysates were assessed. specifically, the protein content of the tissue lysates was determined through a bicinchoninic acid (BCA) protein assay (Thermo Scientific-Pierce, Rockford, IL, United States). The lysis supernatants were adjusted to obtain equal protein concentrations, resolved by SDS-PAGE on precast 10% Tris-glycine gels and transferred to polyvinylidene difluoride membranes (Merck Millipore, Billerica, MA, United States), and the membranes were subsequently blocked for 30 min at room temperature with 5% skim milk-TBST (1 × TBS plus 0.05% Tween-20). After incubation with the primary antibodies under blocking conditions, the proteins were detected with the appropriate secondary antibody (peroxidase-linked anti-rabbit or anti-mouse IgG) and enhanced chemiluminescence (Merck Millipore, Billerica, MA, United States). Antibodies specific for β-actin (1:5000), p35/25 (1:2000, v/v), tau (1:3000, v/v), and p-tau^Ser396^ (1:2000, v/v) were purchased from Cell Signaling Technology (Danvers, MA, United States), and an antibody specific for mPGES1 (1:3000, v/v) was purchased from Santa Cruz Biotechnology (Santa Cruz, CA, United States). An antibody specific for EP1 (1:2000, v/v) was purchased from Abcam (Cambridge, MA, United States), and antibodies specific for EP2 (1:2000, v/v), EP3 (1:2000, v/v), and EP4 (1:2000, v/v) were obtained from Cayman Chemical (Ann Arbor, MI, United States). Each membrane was probed with only one antibody, and β-actin was used as a loading control. All western blot experiments were performed at least in triplicate, and a different cell or tissue preparation was used for each replicate.

### Immunohistochemistry

Mouse brains were collected from 9-month-old WT or APP/PS1 and COX-2 Tg mice and immobilized with 4% paraformaldehyde. Serial 10-μm thick sections were cut on a cryostat (CM1850; Leica, Wetzlar, Germany). The slides were rehydrated in a graded series of ethanol and submerged in 3% hydrogen peroxide to eliminate endogenous peroxidase activity. The levels of mPGES1, EP1-4 and p-tau were determined using an immunohistochemical staining kit according to the manufacturer’s instructions (Invitrogen, Carlsbad, CA, United States). In select experiments, the slices from human and mouse brains were double-stained with p-tau (Ser396) (Alexa Fluor 555-labeled secondary IgG) and mPGES1 (Alexa Fluor 488-labeled secondary IgG) antibodies (Wang et al., 2011, 2016; Yu et al., 2015).

### Lentiviral Vector Preparation

Lentiviral vectors encoding the mouse mPGES1 gene and a control lentiviral vector were provided by Keygen Biotech. Co. (Nanjing, China). Furthermore, the following short hairpin sequences were synthesized and cloned into the lentiviral vectors: mPGES1 shRNA, 5′-GATCCGCCAGCAGCTGAAGCCTCCTCACTCGAGTGAGGAGGCTTCAGCTGCTGGCTTTTTG-3′, and scramble shRNA, 5′-GATCCGCTGAAGGTCGCTTGGTTCAAGAGACCAAGCGACCTCCAGCATCTTTTTTG-3′. The lentiviral vectors were purified and then co-transfected with packaging vectors (psPAX_2_ and PMD_2_G) (Invitrogen, Carlsbad, CA, United States) into HEK293T cells. After 48 h, the lentiviral particles in the supernatant were concentrated by ultracentrifugation and resuspended in PBS(-). For mPGES1 knockdown, the lentiviral particles containing mPGES1 shRNA or scramble shRNA (Santa Cruz, Delaware, CA, United States) were adjusted to 10^6^–10^7^ titers before injection into the ventricles and hippocampus of mice.

### Transfection

For the ectopic expression of NMDAR or mPGES1, mouse primary neurons were transfected with 1.6 μg/dish of plasmid containing the NMDAR or mPGES1 vector. The primers for mPGES1 insertion into plvx-IRES-zsgreen were as follows: F-GGATCTATTTCCGGTGAATTCATGCAGCCTGCTTCTGCAAAGTGGTACGAT and R-GGAGGGAGAGGGGCGGGATCCTTACTCCAGATCTGGCATCTTTTCATCATC. The following primers were used to insert NMDAR into plvx-IRES-zsgreen: F-NNNNTCTAGAATGAGCACCATGCACCTGCT and R-NNNNGGATCCTCAGCTCTCCCTATGACGGGAACAC. In control experiments, the cells were transfected with 1.6 μg/dish of the empty vector.

### Lentiviral Particle Infection

N2a cells were seeded in 24-well plates at a density of 2 × 10^5^ cells/well. Lentiviral particles and 8 μg/ml polybrene (Sigma-Aldrich, St. Louis, MO, United States) were added to the culture, and the mixture was centrifuged for 90 min at 1.5 × 10^3^ rpm. The supernatant was removed immediately after infection and replaced with basal medium (Invitrogen, Carlsbad, CA, United States) containing 10% fetal bovine serum and 50% conditioned medium. After 72 h, the infection efficiency was determined by qRT-PCR and western blotting.

### Lentiviral Particle Injection

Three-month-old C57BL/6 WT mice were anesthetized and fixed on a stereotactic frame. Lentiviral particles (5 μL) were injected into the hippocampus using a Hamilton syringe. For hippocampus injection, stereotactic injections were administered at the following coordinates relative to the bregma: anteroposterior, 2 mm; mediolateral, 1.2 mm; and dorsoventral, -2 mm. Two months later, the effects of mPGES1 on neural impairment were determined.

### Morris Water Maze

After 3 months of treatment with a lentivirus for silencing mPGES1, the mice were trained and tested in a Morris water maze. Briefly, the mice were pretrained in a circular water maze with a visible platform for 2 days. The platform was then submerged inside the maze such that the deck was 0.5 cm below the surface of the water for the following experiments. Two liters of milk was added to the water to hide the platform from sight. The mice were placed inside the maze and allowed to swim freely until they found the hidden platform. The entire experiment lasted for 7 days. For the first 6 days, the mice were left in the maze and allowed to find the platform for at most 60 s. The learning sessions consisted of four trials each day with an interval of 1 h between the sessions. The spatial learning scores (the latency period necessary to find and climb onto the hidden platform and the length of the path to the platform) were recorded. On the last day, the platform was removed, and the amount of time that elapsed before the mice passed through the memorized region (to a maximum of 2 min) was recorded. Finally, the recorded data were analyzed using statistical software.

### Nest Construction

The mice were housed in corncobs for 1 week before the nest construction test. Two hours before the onset of the dark phase of the light cycle, eight pieces of paper (5 × 5 cm^2^) were introduced into the home cage to create conditions for nesting. The following morning, the nests were scored according to the following four-point system: 1, no biting/tearing with random dispersion of the paper; 2, no biting/tearing of the paper with the paper gathered in a corner/side of the cage; 3, moderate biting/tearing of the paper with the paper gathered in a corner/side of the cage; and 4, extensive biting/tearing of the paper with the paper gathered in a corner/side of the cage.

### Animal Management

This study was performed in accordance with the recommendations of the Care and Use of Medical Laboratory Animals (Ministry of Health, Beijing, China). The protocol was approved by the Laboratory Ethics Committee of Northeastern University and China Medical University.

### Human Brain Samples

Human brain samples with the serial numbers P535-00 (normal) and T4304 (an 88-year-old female and an 84-year-old female with severe and end-stage AD, respectively) were obtained from the New York Brain Bank (Columbia University, New York, NY, United States).

### Statistical Analysis

All the data are presented as the means ± S.E. The statistical significance of the differences between the means was determined using Student’s *t*-test, one-way ANOVA or two-way ANOVA, as appropriate. If the means were found to be significantly different, multiple pairwise comparisons were performed with Tukey’s *post hoc* test (Wang et al., 2014a).

## Results

### A High Concentration of Ca^2+^ Is Able to Stimulate Cdk5 Activity and Tau Phosphorylation

**FIGURE 1:**
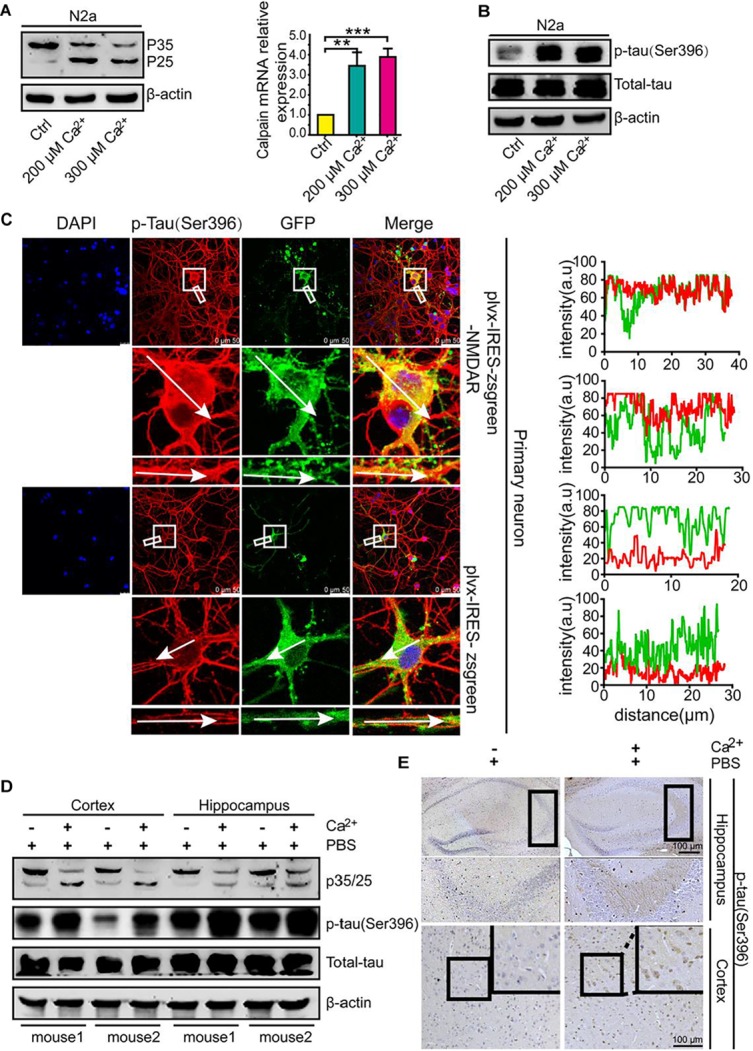
A high concentration of Ca^2+^ is able to stimulate the activity of Cdk5 and tau phosphorylation. **(A,B)** N2a cells were treated with CaCl_2_ (200 or 300 μM) for 24 h. The p-tau levels and the total protein expression levels of tau, p35, and p25 were determined by western blotting using β-actin as an internal control. The mRNA expression of calpain was determined by qRT-PCR using GAPDH as an internal control. **(C)** Primary cultured neuronal cells were transfected with plvx-IRES-zsgreen-NMDAR or empty plvx-IRES-zsgreen plasmids. After 48 h, the transfected cells were immunostained for p-tau^Ser396^ with a primary rabbit p-tau^Ser396^ antibody and an Alexa Fluor 555-labeled goat anti-rabbit IgG secondary antibody (red). The colocalization of p-tau^Ser396^ and NMDAR was semiquantitatively analyzed using ImageJ software. The scale bar represents 50 μm. **(D,E)** CaCl_2_ (3 μg/5 μL) was injected (i.c.v.) into the ventricles of C57BL/6 mice (*n* = 6). After 24 h, the brains were collected and separated into the cerebral cortex and hippocampus, and the p35/25 and p-tau protein levels and the total tau protein levels were determined by western blotting using β-actin as an internal control. The immunoreactivity of p-tau^Ser396^ was determined by immunohistochemistry. The scale bar represents 100 μm. The data represent the means ± S.E. of the independent experiments. ^∗∗^*p* < 0.01 and ^∗∗∗^*p* < 0.001 vs. vehicle-treated controls.

Because p25 is produced and cleaved from p35 to activate Cdk5 and p-tau in p25-overexpressing transgenic mice (Ahlijanian et al., 2000), we evaluated the production of p25 in Ca^2+^-treated N2a cells. The results demonstrated that Ca^2+^ incubation clearly induced the production of p25 (Figure 1A, left panel and Supplementary Figure 1A). Because p25 is truncated from p35 by calpain (Nath et al., 2000), we continued to evaluate the mRNA expression of calpain in Ca^2+^-treated N2a cells. The results demonstrated that mRNA expression was highly induced by treatment with a high concentration of Ca^2+^ (200 and 300 μM) (Figure 1A, right panel). Due to the ability of Cdk5 to phosphorylate tau (Ahlijanian et al., 2000; Liu et al., 2002), experiments were performed to analyze the effects of Ca^2+^ on tau phosphorylation. As expected, Ca^2+^ treatment clearly increased the phosphorylation of tau at Ser 396 (Figure 1B and Supplementary Figure 1A). To further confirm whether the addition of exogenous Ca^2+^ can stimulate the phosphorylation of tau, *N*-methyl-D-aspartate receptor (NMDAR)-overexpressing cells were established as an *in vitro* model for Ca^2+^ influx in primary cultured neurons (Figure 1C). The results demonstrated that Ca^2+^ influx via NMDAR overexpression robustly increased the phosphorylation of tau in the spines of neurons compared with that obtained with Ca^2+^ influx in empty-transfected controls (Figure 1C, left panel). Notably, the intensity analysis also confirmed this result (Figure 1C, right panel). To further validate the *in vitro* observations, CaCl_2_ (3 μg/5 μL) was injected (i.c.v.) into the ventricles of C57BL/6 mice (*n* = 6). After 24 h, the production of p25 and p-tau was analyzed by western blotting. Similarly, the results revealed that Ca^2+^ treatment concurrently increased the phosphorylation of tau and increased the expression level of p25 in the cerebral cortex and hippocampus of mice (Figure 1D and Supplementary Figure 1B). In addition, p-tau (Ser 396) was immunostained with a p-tau-specific antibody. The morphology analysis demonstrated that Ca^2+^ treatment clearly increased the phosphorylation of tau in the cerebral cortex and hippocampus of C57BL/6 mice (Figure 1E). Accordingly, Ca^2+^ was clearly able to stimulate the phosphorylation of tau by increasing the production of p25, an activator of Cdk5 *in vitro* and *in vivo*.

### Cdk5 Activity, Ca^2+^ Concentration and p-Tau Are Elevated in APP/PS1 Tg Mice

**FIGURE 2:**
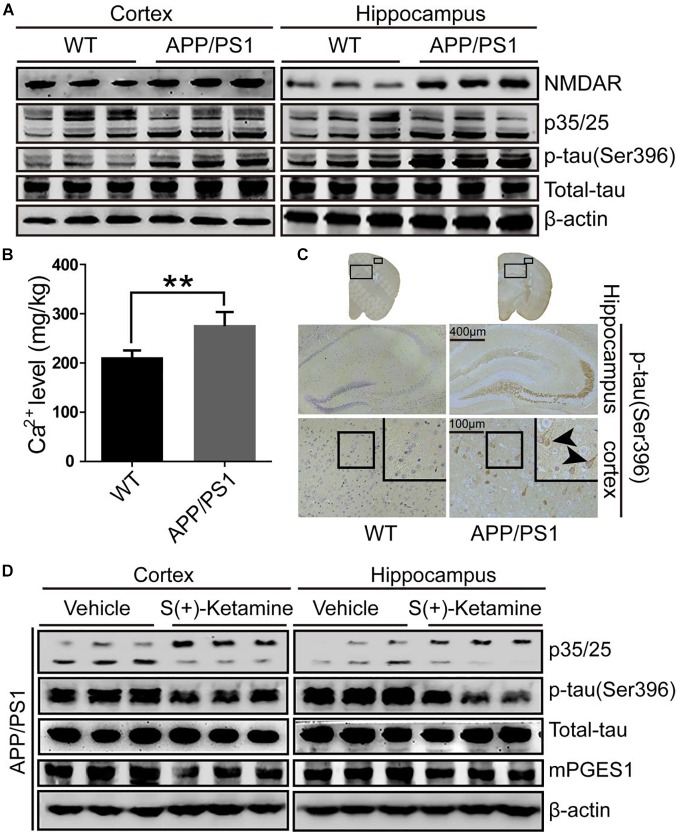
NMDAR is responsible for mediating the effects of Ca^2+^ on stimulating the production of p25 and phosphorylating tau at Ser396. **(A–C)** The brains of 9-month-old APP/PS1 Tg mice were collected after anesthesia and perfusion. **(A)** The NMDAR, p35/25 and p-tau levels and the total protein expression levels of tau were determined by western blotting using β-actin as an internal control. **(B)** The Ca^2+^ concentration was determined by atomic absorption spectroscopy. **(C)** The morphology of p-tau^(Ser396)^ in APP/PS1 Tg mice was determined by immunohistochemistry. The scale bar represents 400 or 100 μm. **(D)** APP/PS1 Tg mice were treated with S(+)-ketamine (50 mg/kg) for 3 months (*n* = 6). The p35/25 and p-tau^(Ser396)^ levels and the total protein expression levels of tau were determined by western blotting using β-actin as an internal control. The data represent the means ± S.E. of independent experiments. ^∗∗^*p* < 0.01 with respect to the WT mice.

Because studies have suggested the pivotal roles of Cdk5 in the pathogenesis of AD (Leclerc et al., 2001), we evaluated the activity of p25, an activator of Cdk5, in the brains of 9-month-old APP/PS1 Tg mice, an AD experimental model (*n* = 6). As shown in Figure 2A and Supplementary Figure 2A, p25 immunostaining was highly enhanced in the cerebral cortex and hippocampus of 9-month-old APP/PS1 Tg mice compared with that in C57BL/6 mice. These data also indicated that Cdk5 activity is upregulated during the development and progression of AD. To further confirm the effect of Cdk5 on tau phosphorylation, we examined the levels of tau phosphorylation in APP/PS1 Tg mice. Consistent with our hypothesis, the phosphorylation of tau was upregulated in both the cerebral cortex and hippocampus of the mice (Figure 2A,C). However, whether Ca^2+^ and its transporter are elevated in APP/PS1 Tg mice, an experimental AD model, remains questionable. Because it has been reported that NMDAR potentially contributes to the transport of Ca^2+^ to neurons (Ngo-Anh et al., 2005), we determined the expression of NMDAR in APP/PS1 Tg mice (*n* = 6). Consistent with our hypothesis, the expression of NMDAR was upregulated in the cerebral cortex and hippocampus of the mice (Figure 2A). Initially, we determined the levels of Ca^2+^ in APP/PS1 Tg mice. As expected, the results demonstrated that the concentration of Ca^2+^ was markedly greater than that in WT mice (Figure 2B). To further determine the key roles of NMDAR in the protein kinase activity of Cdk5 and the phosphorylation of tau, we treated APP/PS1 Tg mice (*n* = 6) with S(+)-ketamine, an NMDAR antagonist. The results showed that S(+)-ketamine significantly reduced the content of p25, the phosphorylation of tau and the expression of mPGES1 (Figure 2D and Supplementary Figure 2B). Based on these observations, Ca^2+^ stimulated the phosphorylation of tau at Ser 396 through an NMDAR-dependent p25-activating mechanism.

### mPGES1 Is the Downstream Target of COX-2 That Induces the Impairment of Neurons by Phosphorylating Tau

**FIGURE 3:**
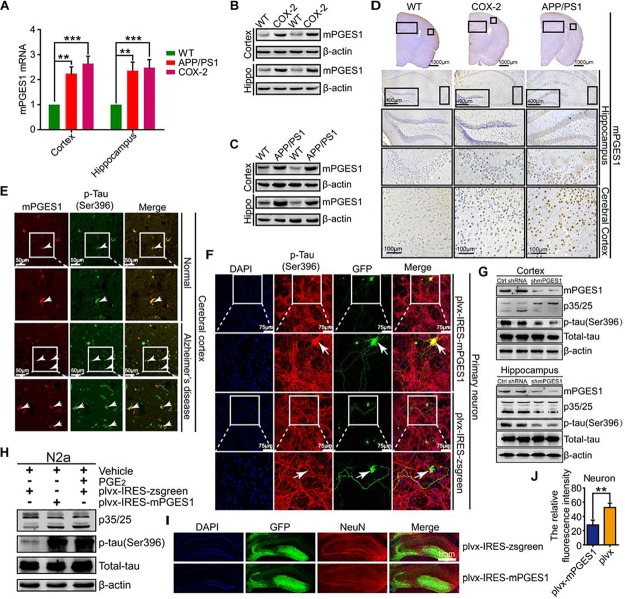
mPGES1 is upregulated in APP/PS1 Tg mice, and this upregulation results in neuronal impairment through the phosphorylation of tau. **(A–D)** The brains of 9-month-old APP/PS1 or COX-2 Tg mice were collected after anesthesia and perfusion (*n* = 6). **(A)** The mRNA expression level of mPGES1 was determined by qRT-PCR using GAPDH as an internal control. **(B,C)** The protein expression level of mPGES1 was determined by western blotting using β-actin as an internal control. **(D)** The morphology of mPGES1 in APP/PS1 and COX-2 Tg mice was determined by immunohistochemistry. The scale bar represents 400 or 100 μm. **(E)** Tissue blocks of human brains were collected by the New York Brain Bank (*n* = 1). Free-floating slices (40 μm) were prepared using a cryostat. The slices were double-stained with mPGES1 (red) and p-tau^Ser396^ (green). The arrows indicate the colocalization of COX-2 and p-tau^Ser396^ (yellow). The scale bar represents 50 μm. **(F)** Primary cultured neurons were transfected with plvx-IRES-zsgreen-mPGES1 or empty plvx-IRES-zsgreen plasmids. After 48 h, the transfected cells were immunostained for p-tau^Ser396^ with a primary rabbit p-tau^Ser396^ antibody and an Alexa Fluor 555-labeled goat anti-rabbit IgG secondary antibody (red). The arrows indicate the colocalization of mPGES1 and expressed tau^Ser396^ (yellow). The scale bar represents 75 μm. **(G)** The brains of APP/PS1 Tg mice were infected with lentivirus containing mPGES1 shRNA (*n* = 6). The protein expression of mPGES1 and p35/25 and the phosphorylation of tau at Ser396 were analyzed by western blotting. **(H)** N2a cells were either transfected with plvx-IRES-mPGES1 or treated with PGE_2_. The p35/25 and p-tau levels and the total protein expression levels of tau were determined by western blotting using β-actin as an internal control. **(I)** The lentiviral particles containing plvx-IRES-zsgreeen-mPGES1 or empty plvx-IRES-zsgreen were injected into the hippocampus of mice (*n* = 6). The neurons were immunostained with a NeuN-specific antibody (red). mPGES1 showed colocalization with NeuN (yellow). The scale bar represents 1000 μm. **(J)** The fluorescence intensity was quantified using ImageJ software. These images shown are representative of six independent mouse experiments. The data represent the means ± S.E. of independent experiments. ^∗∗^*p* < 0.01 and ^∗∗∗^*p* < 0.001 with respect to the WT mice.

We speculated that mPGES1, as the downstream target of COX-2, is involved in the regulation of tau phosphorylation. To investigate this hypothesis, we first determined the expression levels of mPGES1 in COX-2 Tg mice (*n* = 6). The results showed that mPGES1 expression was elevated in the cerebral cortex and hippocampus of COX-2 Tg mice (Figure 3A,B and Supplementary Figures 3A,D). Because COX-2 is stimulated in APP/PS1 mice (Ferretti et al., 2012), we investigated the expression of mPGES1 in AD animal models. As expected, the expression of mPGES1 was highly induced in APP/PS1 Tg mice (Figure 3A,C and Supplementary Figures 3B,D). mPGES1 was also colocalized with phosphorylated tau in the brain slices of AD patients (Figure 3E). To further assess the effect of mPGES1 on the phosphorylation of tau at Ser-396, we established an *in vitro* model of mouse primary neurons overexpressing mPGES1, which is the synthetase of PGE_2_, and found that the overexpression of mPGES1 robustly upregulated the phosphorylation of tau (Figure 3F). To further illustrate the relationship between mPGES1 and tau phosphorylation, we infected the brains of APP/PS1 Tg mice with lentivirus containing mPGES1 shRNA (*n* = 6). The data showed that the production of p25 and the phosphorylation of tau were significantly inhibited in the cerebral cortex and hippocampus of lentivirus-infected mice (Figure 3G and Supplementary Figure 3C), but the mPGES1 does not significantly affect the mRNA expression of EP1-4 in shmPGES1 lentivirus-injected APP/PS1 Tg mice (Supplementary Figure 5). In addition, we transfected N2a cells with mPGES1-overexpressing plasmids or treated the cells with PGE_2_, and the results demonstrated that both transfection with mPGES1 cDNA plasmids and PGE_2_ treatment clearly increased the proportion between p25 and p35 and thereby induced the hyperphosphorylation of tau (Figure 3H). More importantly, mPGES1 was also observed to be an important enzyme that phosphorylates tau in N2a cells (Figure 3H and Supplementary Figure 3D). Although mPGES1 and PGE_2_ were shown to have the ability to phosphorylate tau, their effects on neurons remained in question. Therefore, we injected (i.c.v.) lentivirus containing shmPGES1 into the ventricles of C57BL/6 mice (Figure 3I). The immunofluorescence intensity of neurons was analyzed by immunostaining with NeuN antibody. The results demonstrated that the fluorescence intensity of stained NeuN was greatly suppressed by mPGES1 overexpression (Figure 3J), which suggested that the activity of mPGES1 results in neuronal loss during the course of AD development and progression.

### EP1-3 Receptors Are Critical for Mediating the Effects of PGE_2_ on Phosphorylating Tau by Inducing the Production of p25

**FIGURE 4:**
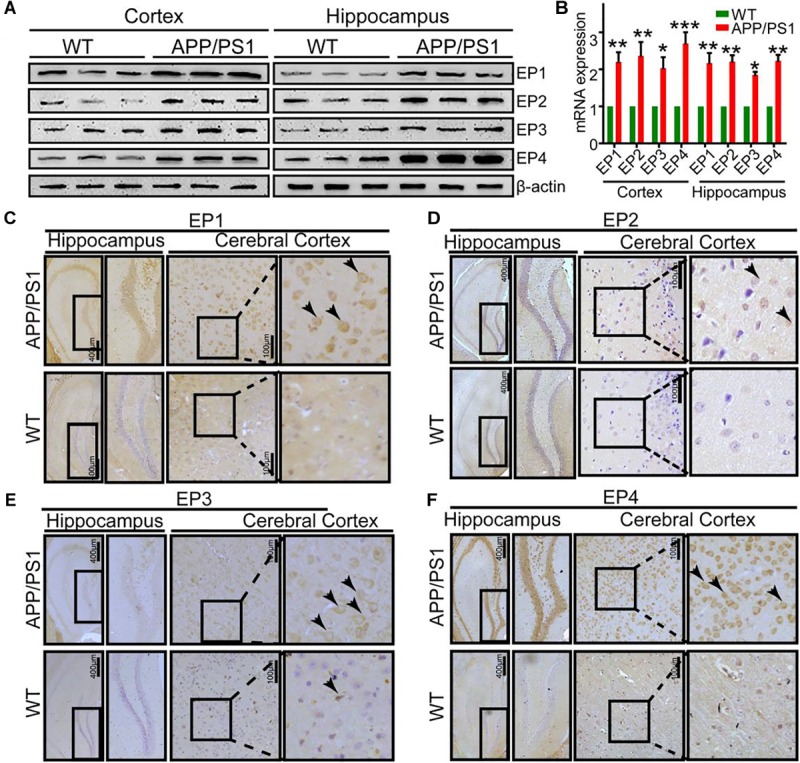
The expression of EPs is upregulated in APP/PS1 Tg mice. **(A–F)** The brains of 9-month-old APP/PS1 Tg mice were collected after anesthesia and perfusion (*n* = 6). **(A,B)** The mRNA and protein expression levels of EP1-4 were determined by qRT-PCR and western blotting, respectively. β-actin and GAPDH served as the internal controls. **(C–F)** The morphology of EP1-4 was determined by immunohistochemistry. The scale bar represents 400 or 100 μm. These images are representative of six independent mouse experiments. The data represent the means ± S.E. of independent experiments. ^∗^*p* < 0.05, ^∗∗^*p* < 0.01, and ^∗∗∗^*p* < 0.001 with respect to the WT mice.

**FIGURE 5:**
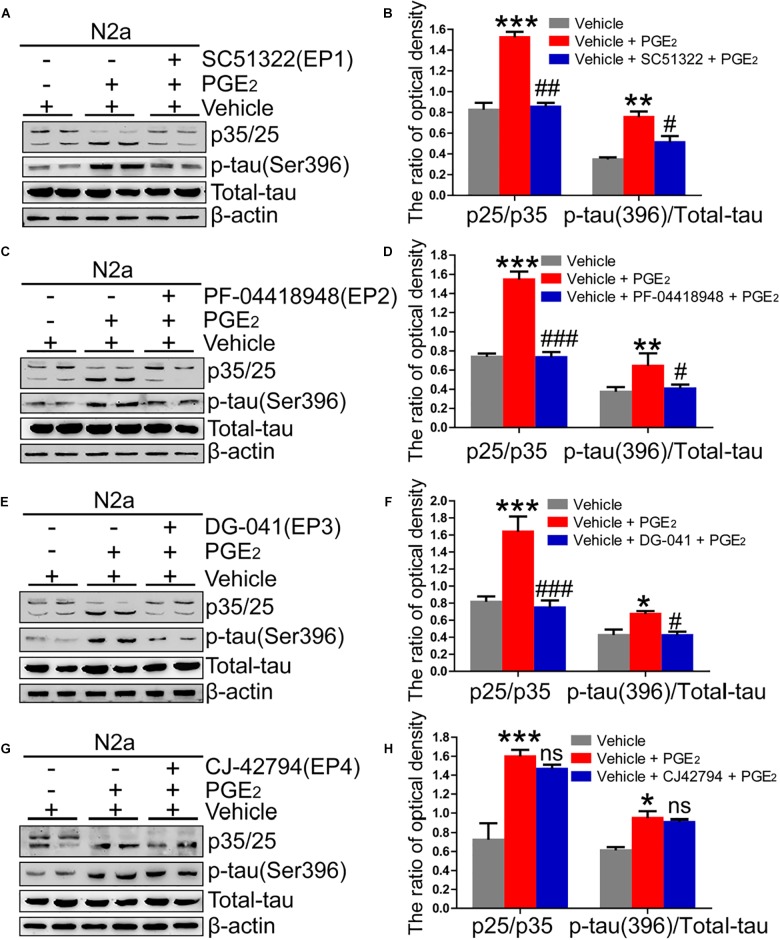
EP1-3, but not EP4, mediates the effects of Ca^2+^ on phosphorylating tau. **(A–H)** N2a cells were treated with PGE_2_ (500 nM) in the absence or presence of EP1-4 inhibitors (SC51322, antagonist of EP1; PF-04418948, antagonist of EP2; DG-041, antagonist of EP3; and CJ-42794, antagonist of EP4) for 24 h (*n* = 6). The expression of phosphorylated tau and p35/25 and the total protein expression level of tau were determined by western blotting using β-actin as an internal control. The data represent the means ± S.E. of independent experiments. ^∗^*p* < 0.05, ^∗∗^*p* < 0.01 and ^∗∗∗^*p* < 0.001 compared with the vehicle-treated control. ^#^*p* < 0.05, ^##^*p* < 0.01, and ^###^*p* < 0.001 compared with the PGE_2_-treated group.

Due to the ability of PGE_2_ to phosphorylate tau, we speculated that EPs, which are PGE_2_ receptors, are also involved in regulating the phosphorylation of tau. To test this hypothesis, we initially determined the expression of EPs in APP/PS1 Tg mice. The results demonstrated that EPs were highly expressed in these Tg mice (Figure 4A–F and Supplementary Figure 4A). To further verify the roles of EPs in tau phosphorylation, antagonists were used to inhibit EP activities in N2a cells. Consequently, we observed that the inhibition of EP1-3, but not EP4, clearly decreased the phosphorylation of tau by restoring the proportion between p25 and p35 (Figure 5A–H). Therefore, the mPGES1, PGE_2_, and EP1-3 pathways are critical for mediating the effects of Ca^2+^ on stimulating the phosphorylation of tau by inducing p25 during AD development and progression.

### The Knockdown of mPGES1 Ameliorates Cognitive Decline in APP/PS1 Tg Mice

**FIGURE 6:**
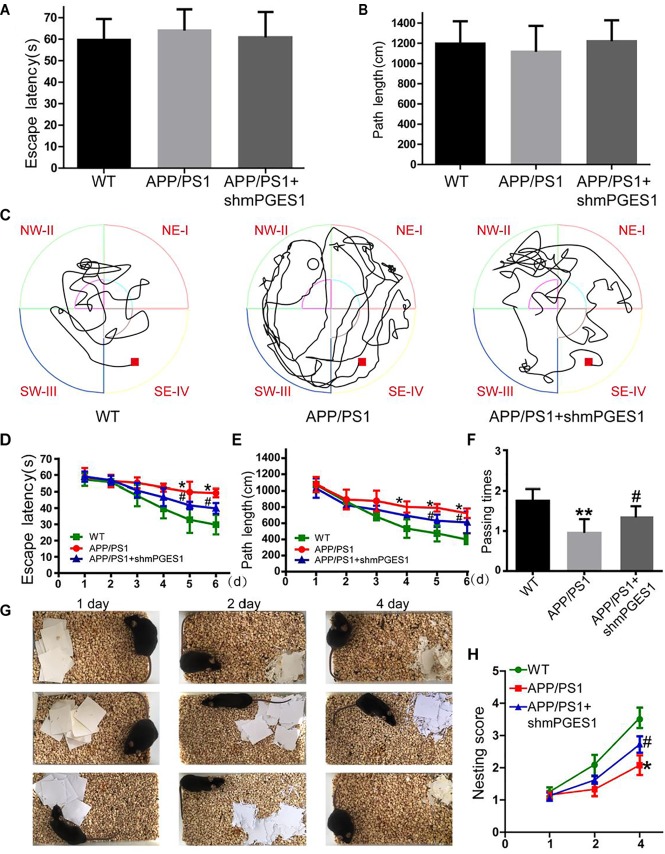
The learning ability is improved by the silencing of mPGES1 in APP/PS1 Tg mice. Three-month-old APP/PS1 Tg mice were treated with lentivirus containing shRNA targeting mPGES1, and 6 months later, their learning ability was evaluated. **(A,B)** The cognitive ability of the mice was evaluated using the Morris water maze test. **(C–E)** Hidden-platform tests. **(F)** In the probe trial on day 7, the mice in the APP/PS1 group showed the fewest number of passes through the former location of the platform, and the lentivirus-treated group exhibited partially reversed effects of APP/PS1 damage and improved cognition and memory abilities. **(G,H)** The nest construction abilities of 9-month-old WT, APP/PS1 Tg mice, and APP/PS1 Tg mice treated with shmPGES1 were quantified (*n* = 6). Each group was tested six times. ^∗^*p* < 0.05 and ^∗∗^*p* < 0.01 compared with the control group; ^#^*p* < 0.05 compared with the APP/PS1 Tg mice.

Considering the observation that mPGES1 is involved in aggravating tau hyperphosphorylation, we investigated the relationship between the brain mPGES1 expression levels and memory deficits in APP/PS1 Tg mice (*n* = 6). Treatment with lentivirus containing shmPGES1 was initiated at 3 months of age. After 6 months of shmPGES1 treatment, we assessed the spatial learning and memory abilities of the mice using the Morris water maze task. The pretraining results from the visible platform tests with the APP/PS1 and lentivirus-treated APP/PS1 mice did not differ from those obtained with the WT C57BL/6 control mice (Figure 6A,B), which suggested that shmPGES1 administration did not significantly influence the motility or vision of the C57BL/6 mice. The untreated APP/PS1 Tg mice exhibited unequivocal learning deficits in this task at 9 months of age, and the lentivirus-treated APP/PS1 Tg mice performed similarly to the WT mice (Figure 6C–E). A probe test performed 24 h after the final training trial revealed that the untreated APP/PS1 mice exhibited no preference toward the target quadrant, which indicated significant memory impairment, whereas the lentivirus-treated APP/PS1 mice performed similarly to the WT C57BL/6 mice (Figure 6F). Moreover, nest construction is an affiliative social behavior. Nest construction was progressively impaired in APP/PS1 Tg mice, but this impairment was reversed by lentivirus treatment for 6 months (Figure 6G,H). These observations further emphasize the pivotal roles of mPGES1 in regulating the development and progression of AD.

## Discussion

**FIGURE 7:**
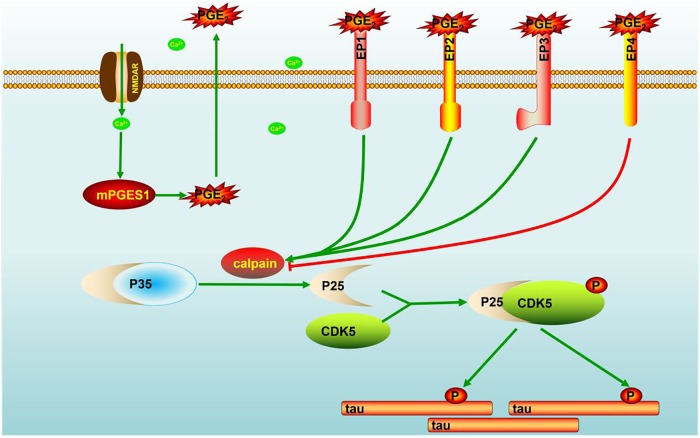
Signaling events of Ca^2+^ in stimulating the phosphorylation of tau by activating mPGES1. During the course of AD development and progression, Ca^2+^ was highly increased in the brains of AD patients and APP/PS1 Tg mice. The highly accumulated Ca^2+^ stimulates the expression of mPGES1 via its transporter, NMDAR. As the synthase of PGE_2_, mPGES1 is responsible for upregulating the expression of calpain, and calpain expression triggers the production of p25, leading to the activation of Cdk5. The activation of Cdk5 ultimately phosphorylates tau at Ser396, and tau phosphorylation results in the cognitive decline of APP/PS1 Tg mice.

Evidence that has accumulated over more than two decades indicates that Ca^2+^ is dysregulated in aging brains and brains with AD, which suggests that Ca^2+^ is involved in the induction of AD (LaFerla, 2002). Consequently, elevated levels of Ca^2+^ exert effects on the phosphorylation of tau (Pierrot et al., 2006). In addition to Ca^2+^, the immunoreactivity of COX-2 is also reportedly increased in neurons in Alzheimer’s disease (AD) brain tissues compared with control brain tissues (Pasinetti and Aisen, 1998). However, the mechanism related to the Ca^2+^ regulation of mPGES1 expression and the relationship between mPGES1 expression and tau phosphorylation have not been elucidated. Therefore, the current investigation aimed to decipher the roles of Ca^2+^ in inducing tau phosphorylation by activating mPGES1 signaling. Considering the critical role of tau phosphorylation in the progression of AD (Ballatore et al., 2007), we sought to identify the mechanisms through which Ca^2+^ regulates tau phosphorylation in APP/PS1 Tg mice. Consequently, we observed that Ca^2+^ treatment specifically activated the signaling pathways of mPGES1 and PGE_2_ via NMDAR (Figure 1C). In addition, the results showed that highly accumulated PGE_2_ regulated the phosphorylation of tau via EP1-3, but not EP4, through a p25-dependent Cdk5-activating mechanism (Figure 5). Finally, p-tau was shown to result in the cognitive decline of AD (Figure 7).

To investigate the roles of Ca^2+^ in regulating the development of AD, we first showed the concentration of Ca^2+^ in the brains of APP/PS1 Tg mice. The results demonstrated that the Ca^2+^ levels were significantly upregulated in the brains of APP/PS1 Tg mice compared with WT mice (Figure 1B). In agreement with our observation, Zhang et al. (2017) previously reported that the Ca^2+^ concentration was markedly increased in the brains of AD patients and APP/PS1 Tg mice. Therefore, a reduction in the release of Ca^2+^ exerts a neuroprotective effect (Stutzmann, 2007). However, we were only able to elevate the Ca^2+^ concentration in the brains of C57BL/6 mice because it is impossible to accurately decrease the concentration of Ca^2+^ to physiological levels during the course of AD development. For the acute experiments, 3 μg/5 μL Ca^2+^ was used for intracerebroventricular (i.c.v.) injection. After Ca^2+^ administration, we observed that Ca^2+^ treatment (3 μg/5 μL) for 24 h increased the phosphorylation of tau by producing p25 (Figure 1D). Consistent with this observation, Ca^2+^ was responsible for the phosphorylation of tau by inducing the production of p25 in acutely injured neuronal cells (Nath et al., 2000). In addition, altered calcium homeostasis might represent a pivotal upstream event in tau phosphorylation (Hartigan and Johnson, 1999). In addition, chronically increased levels of Ca^2+^ are functionally linked to the major features and risk factors of AD, such as tau hyperphosphorylation (Stutzmann, 2007). Although these prior studies have indicated the potential roles of Ca^2+^ in phosphorylating tau, the mechanism has not been elucidated. Moreover, this type of investigation is quite limited because most studies have focused on the roles of Ca^2+^ in the production and deposition of Aβ (Isaacs et al., 2006). To this end, we extended the prior studies to the Ca^2+^ transporter, NMDAR. The NMDAR selective competitive antagonists enhance synaptic plasticity (Dudek and Bear, 1992) and improve cognitive decline in rats (Morris et al., 1986). In agreement with this observation, we also found that NMDAR mediated the effects of Ca^2+^ on the phosphorylation of tau in APP/PS1 Tg mice (Figure 1C,D). Therefore, we elaborated on previous studies to reveal the mechanism of Ca^2+^ in the phosphorylation of tau in APP/PS1 Tg mice.

Because Ca^2+^ has the ability to activate COX-2 (Choudhary et al., 2004; Ogata et al., 2006; Wang J.Y. et al., 2012), we continued to investigate the involvement of COX-2 in mediating the effects of Ca^2+^ on tau phosphorylation. Indeed, several studies have indicated that the neuronal expression of COX-2 is upregulated in AD, which suggests a role for COX-2 in AD pathogenesis (Ho et al., 2001), Our data further showed that COX-2 accelerated the phosphorylation of tau in an mPGES1-dependent manner (Figure 3). In human neurons, microglial cells and astrocytes, mPGES-1 is constitutively expressed and upregulated in AD (Chaudhry et al., 2008). Although no additional studies have shown the ability of mPGES1 to phosphorylate tau, Lee et al. (1999) showed that PGE_2_, which is the metabolic product of mPGES1, increases the gene expression level and production of APP *in vitro*. Additionally, mPGES1 knockdown in Tg2576 mice noticeably disrupts the formation of APs (Akitake et al., 2013). All of these studies indicate that mPGES1 is critical for the onset of AD. More closely, PGI2 and PGF2α affect the phosphorylation of tau in tau^P301S^ Tg mouse models (Wang et al., 2017). Considering these previous studies, our data elaborated on these previous studies and showed that mPGES1 is critical for tau phosphorylation during the course of AD development and progression.

Considering the critical role of mPGES1 in tau phosphorylation, PGE_2_ should also effectively phosphorylate tau because it is the metabolic product of mPGES1. As expected, our data demonstrated that PGE_2_ has the ability to induce the phosphorylation of tau (Figure 5). In support of our data, a previous study showed that the concentration of PGE_2_ is positively associated with the pathogenesis of AD (Montine et al., 1999). Although we did not find additional evidence showing the relationship between PGE_2_ and tau phosphorylation, its upstream enzyme, COX-2, is associated with the formation of NFTs in patients with Fukuyama-type congenital muscular dystrophy (Oka et al., 1999). Moreover, 15d-PGJ_2_, another metabolic product of COX-2, affects tau cleavage, which results in NFT formation in neurodegenerative diseases (Arnaud et al., 2009). Therefore, the involvement of PGE_2_, which is the metabolic product of mPGES1, in mediating the effects of Ca^2+^ on stimulating the phosphorylation of tau was identified in the current study.

Signals of PGE_2_ are typically regarded to be mediated by PGE_2_ receptors, including EP1-4 (Andreasson, 2010), Therefore, experiments were performed to determine the roles of EPs in tau phosphorylation. As a first step, we revealed that the expression of EP1-4 was elevated in the brains of APP/PS1 Tg mice compared with WT mice (Figure 4). In agreement with our data, a previous study showed that EP1-4 is upregulated in APP/PS1 Tg mice (Maingret et al., 2017). Moreover, the results demonstrated that EP1-3, but not EP4, mediated the effects of PGE_2_ on the phosphorylation of tau. To the best of our knowledge, although there is no additional evidence showing the above relationship, EP1-knockdown neurons show resistance to the toxicity of Aβ (Zhen et al., 2012). In addition, EP2 knockdown can improve the cognitive decline of APP23 mice (Hoshino et al., 2012). Based on this observation, Shie et al. (2005) further proposed microglial EP2 as a therapeutic target of AD. EP3 predominantly regulates the proinflammatory signaling pathway at the early stage of AD (Shi et al., 2012). A previous study showed that blocking the activity of EP3 ameliorates the effects of PGE_2_ in impairing hippocampal presynaptic plasticity and identified EP3 as a potential therapeutic target for AD (Maingret et al., 2017) and another study showed that EP3 is critical for the phosphorylation of tau (Guan and Wang, 2018). In contrast, our conclusion is consistent with the observation that the EP4 receptor exerts anti-inflammatory effects *in vitro* and *in vivo* by suppressing the expression of proinflammatory genes in response to LPS treatment, which indicates that the EP4 receptor likely functions as a beneficial receptor *in vivo* against inflammatory diseases of the CNS (Shi et al., 2010). Therefore, we elaborated on this previous study by showing that EP1-3, but not EP4, are involved in regulating the phosphorylation of tau, which results in cognitive decline in AD.

## Ethics Statement

This study was carried out in accordance with the recommendations of the Care and Use of Medical Laboratory Animals (Ministry of Health, Beijing, China). The protocol was approved by the Laboratory Ethics Committee of Northeastern University and China Medical University.

## Author Contributions

L-LC conceived and performed all the experiments. P-PG and Y-YL carried out select the experiments. PW (along with X-SH) conceived the experiments of this study, interpreted the data and wrote the manuscript.

## Conflict of Interest Statement

The authors declare that the research was conducted in the absence of any commercial or financial relationships that could be construed as a potential conflict of interest.
