# Supplementary material for: Calcium Ions Stimulate the Hyperphosphorylation of Tau by Activating Microsomal Prostaglandin E Synthase 1
Source: Front Aging Neurosci. 2019 May 9;11:108. doi: 10.3389/fnagi.2019.00108 (PMC6521221; doi:10.3389/fnagi.2019.00108)
Supplement: Supplementary file 1 [file Data_Sheet_1.docx]

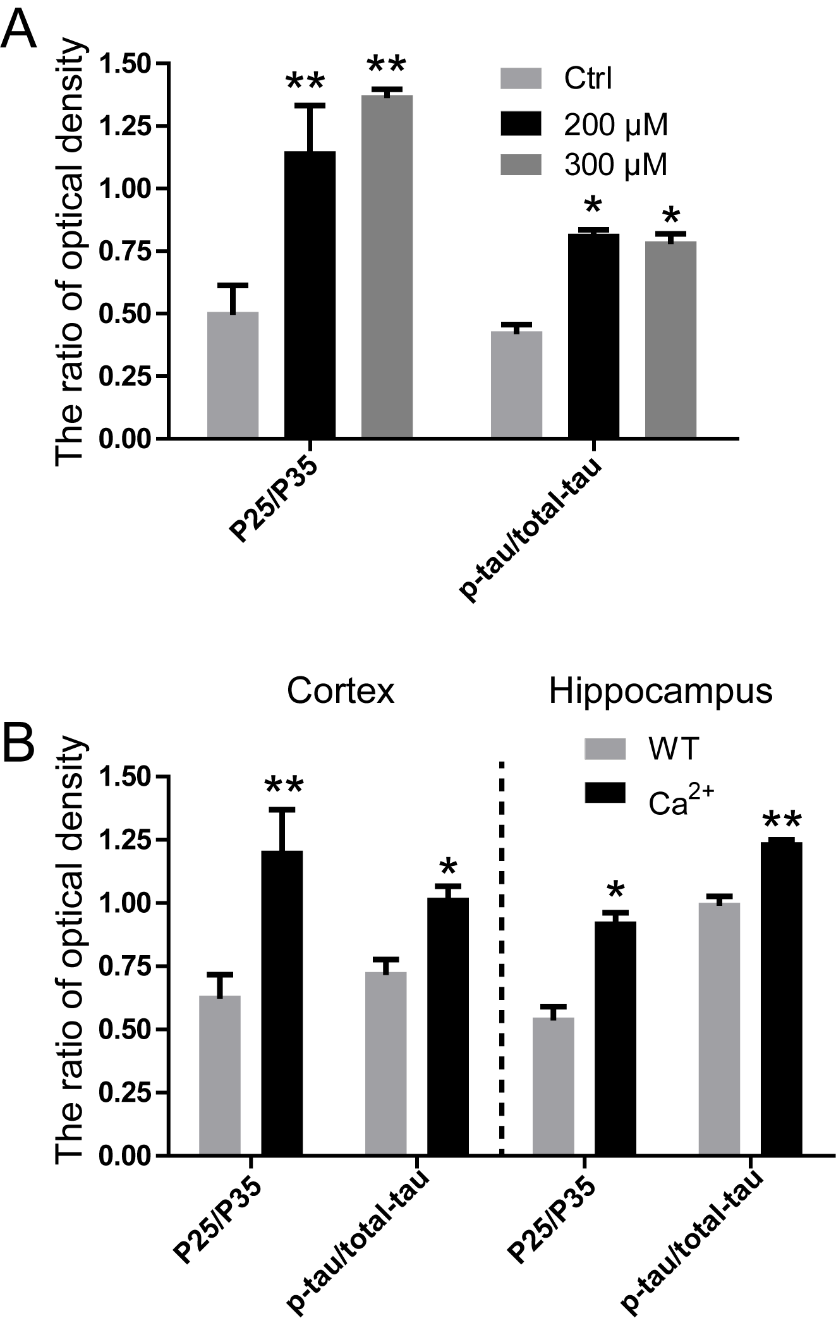


**Supplemental Figure 1. The bands of western blots in Figure 1A, B (A) and Figure 1D (B) were analyzed by Image J.**


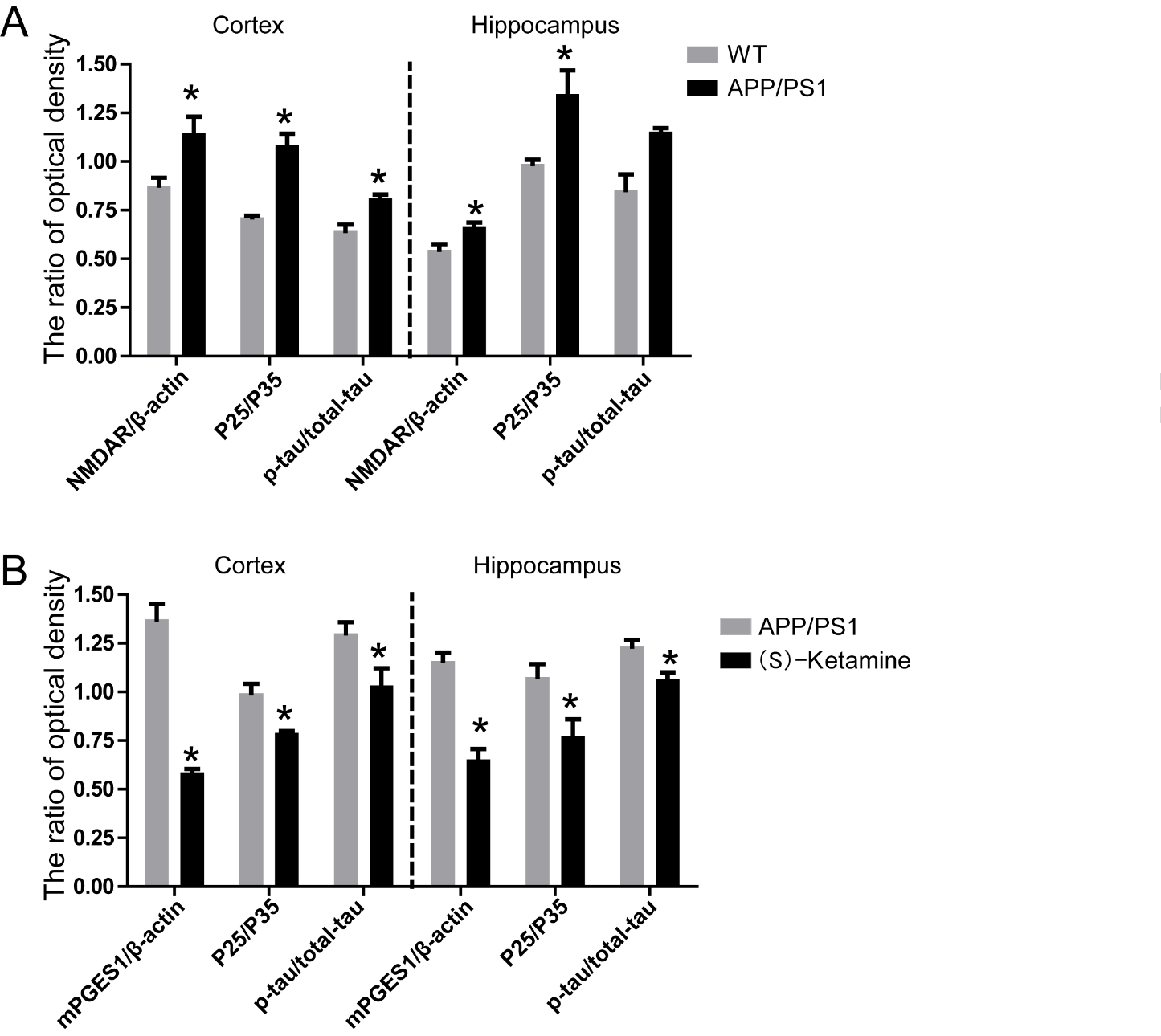


**Supplemental Figure 2. The bands of western blots in Figure 2A (A) and Figure 2D (B) were analyzed by Image J.**


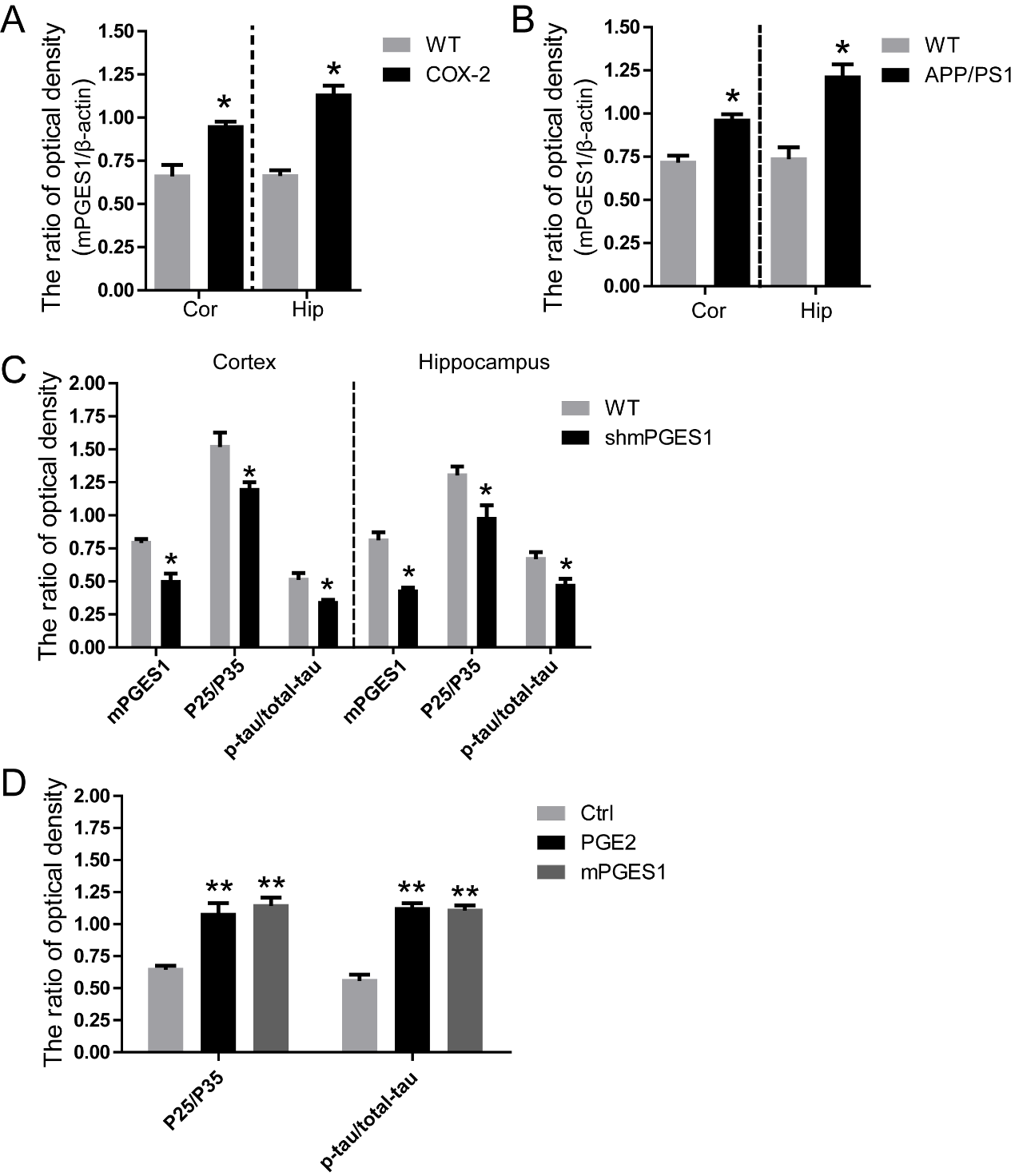


**Supplemental Figure 3. The bands of western blots in Figure 3B (A) , Figure 3C (B), Figure 3G (C) and Figure 3H (D) were analyzed by Image J.**


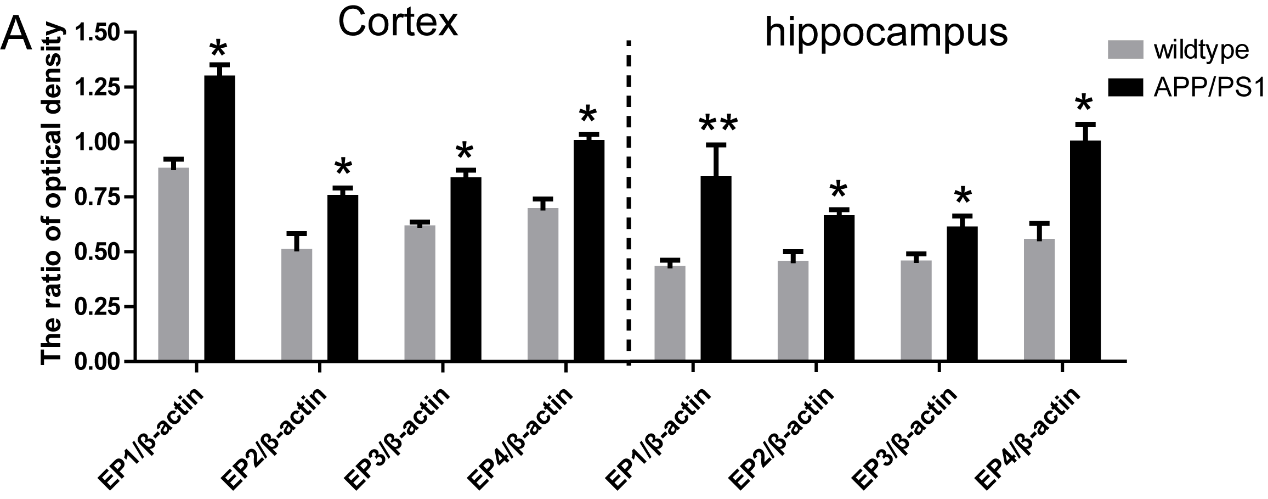


**Supplemental Figure 4. The bands of western blots in Figure 4A (A) were analyzed by Image J.**


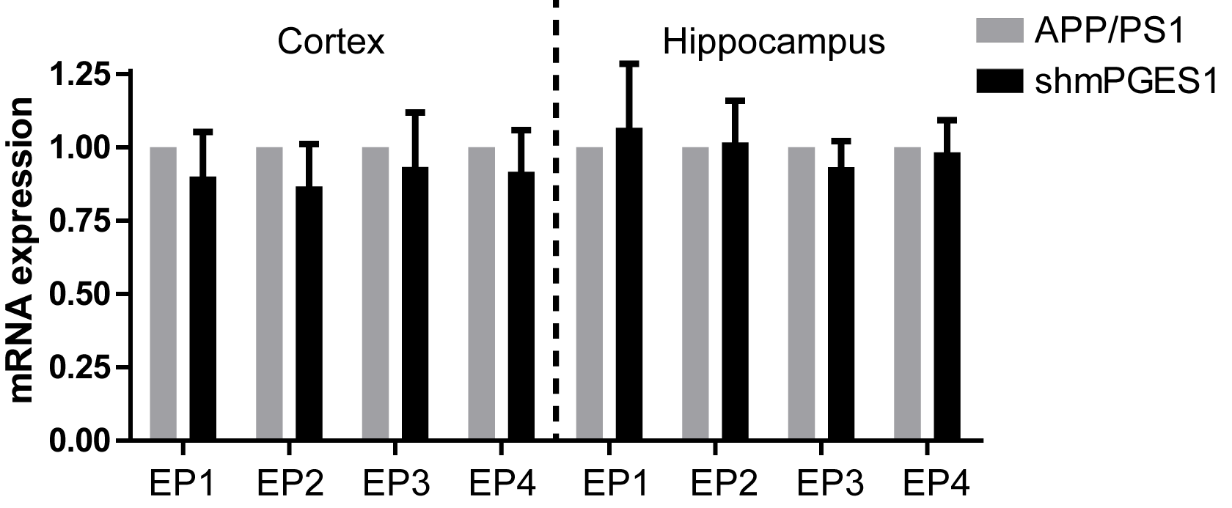


**Supplemental Figure 5. mPGES1 does not affect the mRNA expression of EP1-4 in shmPGES1 lentivirus-injected APP/PS1 Tg mice.** The APP/PS1 Tg mice were injected (i.c.v) with concentrated lentivirus of mPGES1 shRNA. After 6 months, the cortex and hippocampus were collected and extracted total mRNA. The mRNA expression of EP1-4 was determined by qRT-PCR. Even though the average mRNA expression of EP1-4 were suppressed by mPGES1 shRNA in the cortex of mice, there are not statistically significant.
